# Metagenomic Analysis of Dental Plaque on Pit and Fissure Sites With and Without Caries Among Adolescents

**DOI:** 10.3389/fcimb.2021.740981

**Published:** 2021-10-27

**Authors:** Liangyue Pang, Yinuo Wang, Yun Ye, Yan Zhou, Qinghui Zhi, Huancai Lin

**Affiliations:** ^1^Department of Preventive Dentistry, Hospital of Stomatology, Sun Yat-sen University, Guangzhou, China; ^2^Guangdong Provincial Key Laboratory of Stomatology, Guanghua School of Stomatology, Sun Yat-sen University, Guangzhou, China

**Keywords:** dental caries, microbiome, biofilms, bioinformatics, biomarkers

## Abstract

Caries is one of the most prevalent infectious diseases worldwide and is driven by the dysbiosis of dental biofilms adhering to tooth surfaces. The pits and fissured surfaces are the most susceptible sites of caries. However, information on the taxonomic composition and functional characteristics of the plaque microbiota in the pit and fissure sites is very limited. This study aimed to use metagenomic sequencing analyses to investigate the relationship between the plaque microbiome in the pit and fissure site and caries in adolescents. A total of 20 adolescents with active pit and fissure surface caries were involved as well as 20 age-matched, caries-free teenagers for control tests. Plaque samples were collected from the pit and fissure site and were subjected to metagenomic analyses, in which the microbial communities were investigated. Our results showed that the microbiota diversity was similar between those two groups. At the species level, the relative abundances of *A. gerencseriae*, *P. acidifaciens*, *P. multisaccharivorax*, *S. oralis*, *S. mutans*, and *P. denticolens* were higher in the caries-active group. *N. elongata*, *C. hominis*, and *A. johnsonii* were relatively more abundant in the caries-free groups. Functional analysis suggested that the metabolic pathway was the most abundant pathway, and the functional traits of the level 2 pathways included amino acid metabolism, metabolism of cofactors, and vitamins and carbohydrate metabolism. Our results also revealed that the caries group displayed several alterations in metabolic pathways, including enriched functions in carbohydrate digestion and absorption. This study suggested that in addition to the specific anatomical structures of the pit and fissured surfaces, the fundamental differences in the plaque microbiome may also be related to the susceptibility of pit and fissure caries.

## Introduction

As an infectious and chronic disease, dental caries is one of the most prevalent diseases in the world. In China, the 4th National Oral Health Survey reported that the prevalence of permanent caries in 12-year-old children increased during the past decade, from approximately 28.9% to 38.5% (Quan et al., 2018), and 83.4% of decayed permanent teeth remained untreated (Quan et al., 2018). Progressing caries may lead to pulpitis and cause severe pain (Selwitz et al., 2007), which influences children’s class attendance and performance (Jackson et al., 2011). Therefore, caries remains a major health problem for teenagers.

Dental caries is caused by dysbiosis of the plaque biofilm adherent to the tooth surface that creates an acidic microenvironment under excessive exposure to dietary carbohydrates that demineralizes the enamel, leading to permanent damage to the tooth (Pitts et al., 2017). Historically, *mutans streptococci* (*S. mutans*) and *Lactobacillus* species have been considered to be the main pathogens relevant to caries (Kutsch and Young, 2011). However, caries can be observed without detecting these bacteria, indicating that these species cannot sufficiently explain all cases of caries. With the advanced and high-throughput sequencing technologies applied, a growing number of caries-related microbiota have been identified (Nyvad et al., 2013). It is now understood that caries is caused by dysbiosis of the microbiota that colonizes tooth surfaces, and most of them are aciduric and acidophilic bacteria (Nyvad et al., 2013).

With the rapid development of next-generation sequencing techniques, more and more studies analyze the microbiome associated with dental caries. A recently published study reported that *Abiotrophia*, *Prevotella*, and *Veillonella* were found at significantly higher levels in caries lesions of adolescents and *Rothia* and *Corynebacterium* at significantly higher levels in caries-free individuals. (Havsed et al., 2021). Gross et al. compared the bacterial community profiles of adolescents with severe dental caries and healthy controls and concluded that an overall loss of community diversity occurred with the progression of dental caries. Level of *Lactobacillus* species, *S. mutans*, and *Propionibacterium* FMA5 significantly increased with the caries progression (Gross et al., 2010). Most studies are based on pooled samples instead of site-specific samples. Dental caries is a localized disease of teeth (Fejerskov et al., 2008), and carious processes are the combined effect of local microorganisms and environmental factors (Fejerskov et al., 1994). The microbiome may vary significantly over very minor distances (Simon-Soro et al., 2013), and the pooling of plaque samples may make the information obtained from molecular studies of caries unclear. Therefore, studying caries with site-specific biofilm samples is essential. Pits and fissures are the main sites at risk of dental caries, and it is easy to facilitate the structuration and accumulation of oral biofilms. However, a thorough understanding of the special taxa involved in pit and fissure caries has not been achieved. Therefore, the purpose of the present study was to compare the plaque microbial composition and the functional profile in the pit-and-fissure site with active pit-and-fissure caries and caries-free adolescents using a metagenomic sequencing method.

## Materials and Methods

### Subject Recruitment and Sample Collection

The study was authorized by the Ethical Committee of the Hospital of Stomatology, Sun Yat-sen University, in Guangzhou, China (KQEC-2021-24-02). Adolescents with caries and healthy controls were recruited from Xingtan Liangqiuju Junior Middle School in Foshan, Guangdong Province, China. Caries experience was measured as decayed, missing, and filled teeth (DMFT). Their diagnoses were made by a dentist according to the International Caries Detection and Assessment System II (ICDAS-II). Individuals with lesions scoring 3–6 were classified into the caries group. In total, 40 adolescents (aged 12 to 13 years) were involved in the study, including 20 adolescents with more than one pit-and-fissure active caries (DMFT≥1) (Ortiz et al., 2019) and 20 caries-free (DMFT= 0) controls. The exclusion criteria of subjects were as follows: (1) adolescents who used antibiotics within the last 3 months; (2) adolescents infected with bacteria or viruses in other parts of the body; and (3) adolescents with gingivitis or orthodontic appliance or have carried out professional dental prophylaxis, such as pit and fissure sealing. Under the guidance of their guardians, the adolescents completed an oral health questionnaire, consisting of six questions: sex, age, frequency of tooth brushing, toothpaste containing fluoride or not, frequency of snack consumption, and sweet drink consumption. We obtained a statement of informed consent from the parents or legal guardians of all participants.

To collect more amount of dental plaque for metagenomic analysis, we sampled the dental plaque from the same 16 pit and fissure sites (14, 15, 16, 17, 24, 25, 26, 27, 34, 35, 36, 37, 44, 45, 46, 47). Each plaque sample was obtained by pooling from multiple teeth. In the caries group, we sampled the plaque microbiome from pit-and-fissure sites that have not been affected by caries. In other words, the samples in the caries group were collected from the healthy pit-and-fissure sites. Participating adolescents were required to stop brushing their teeth for 12 h, and breakfast was not allowed before the sample collection. Before the examination, all the teenagers were asked to rinse their mouths. First, the teeth were dried with a gentle air stream to avoid saliva contamination before sampling. The dental plaque was removed from each pit and fissure site with a sharp-pointed sterile dental explorer, and the plaque was wiped onto a spoon excavator and then immediately placed in a sterile 1.5-ml Eppendorf tube. The samples were transported to the laboratory as soon as possible and frozen at −80°C before analysis.

### DNA Extraction, Library Preparation, and Illumina NovaSeq Sequencing

The CTAB method was used to extract Microbial DNA from each sample. First, the dental plaque samples were transferred to a 2.0-ml Eppendorf tube containing 1000 μl of CTAB extraction buffer, which was incubated at 65°C and gently mixed for adequate homogenization. After centrifugation, 500 μl of the supernatant was transferred to a fresh tube with an equal volume of phenol chloroform isoamyl alcohol (25:24:1), mixed gently, and centrifuged at 12,000 rpm for 10 min. Then, chloroform isoamyl alcohol (24:1) was added to the supernatant, vortexed for 1 min, and centrifuged at 12,000 rpm for 10 min. Next, 500 μl of the supernatant was transferred to a fresh tube with isopropyl alcohol, mixed by inversion, and incubated at −20°C overnight. The next day, the solution was centrifuged at 12,000 rpm for 10 min and then washed in 75% ethanol twice. The resulting pellet was resuspended in 50 μl of TE buffer (10 mM Tris, 1 mM EDTA). One microliter of RNase A was added to the tube to digest RNA and then incubated at 37°C for 15 min (Gnat et al., 2017). The concentration and purification of the final DNA were detected by Nanodrop and agarose gel electrophoresis.

The NEB Next^®^ Ultra ™ DNA Library Prep Kit (NEB, USA) was used to construct the DNA libraries following the manufacturer’s protocol. Then, the qualified libraries were sequenced with an Illumina NovaSeq PE150 platform at Wekemo Tech Co., Ltd. Shenzhen China. We prepared the samples according to standard protocols. Blank controls were used during DNA extraction and library construction. All the samples in this study were not contaminated.

### Preprocessing of Metagenome Data

The acquired raw reads were processed at Wekemo Tech Co. using Knead Data. Illumina adaptors, 5′ or 3′ bases with quality scores lower than 20, and DNA sequences shorter than 50 bp were trimmed using cutadapt. Each sample was sequenced, with an average of 6.54G sequence read depth per sample. Human reads were then deleted from the metagenomic dataset by aligning DNA reads to the human genome using Bowtie2. Finally, clean data were obtained by removing host-originated reads and low-quality reads.

### Taxonomy Prediction and KEGG Functional Database Annotations

To characterize the taxonomic composition of the metagenomic dataset, kraken2 and self-built database (Wekemo Tech Co.) were used to annotate and classify the clean sequences of all samples and Bracken was used to predict the actual relative abundance of species. According to the algorithms of the lowest common ancestors (Huson et al., 2011) and gene abundance calculations, we gained information at seven taxonomical hierarchies (from the kingdom to species levels) from each sample.

The unigene sequences were aligned against the UniRef90 protein database using DIAMOND (version 0.7.10.59) with default parameters, and the failed reads were filtered out. According to the Uniref90 ID and the corresponding relationship of the KEGG database, the gene abundance table was obtained, and then the functional abundance profiles of each sample were drawn.

### Data Visual Exhibition, Statistical, and Bioinformatics Analysis

R software (version 3.8) and QIIME (version 2) were used to carry out the graphics and statistical analyses in our study. The Dunn test was performed to detect significant differences in relative abundance of different groups, and differences were considered significant when *p* < 0.05. The biomarkers of different groups were defined by LEfSe analysis to identify potential bacteria related to active caries and caries-free caries. The threshold on the logarithmic LDA score was set to 3.0. Venn diagram was used to illustrate the core microbiome at the species level. Non-metric multi-dimensional scaling (NMDS) and principal coordinate analysis (PCoA) based on the Bray–Curtis distance were used to perform the diversity analysis. Multivariate Analysis by Linear Models (MaAsLin) was performed to identify significant associations between plaque microbiome (at species level) and clinical variables. Permutational multivariate analysis of variance (PERMANOVA) was applied to quantify the proportion of variation in taxonomy and pathway profiles explained by caries status and potential cofounding variables. The LEfSe analysis was using to perform Community functional structure comparison, and the logarithmic LDA score cutoff was set to 2.0. The meta-community cooccurrence network was constructed based on correlation coefficients >0.6 and *p* < 0.05.

## Results

### Characteristics Between the Two Groups

In total, plaque samples of the pit and fissure sites were collected from 20 teenagers with pit-and-fissure caries (DMFT ≥ 1) and 20 healthy control subjects (DMFT = 0). As reported in **Supplementary Table 1**, no significant differences were found in clinical data that can be confounding variables between the two groups. After removing the low-quality data and filtering out host contamination, a total of 756142470 (261.7 GB) reads of high-quality sequences were acquired from 40 samples, with 6.54 GB of clean data per sample on average. In total, 2,897 species, 957 genera, and 22 phyla were identified in this study.

### Microbial Characterization and Comparison Between Groups

#### Microbial Richness and Diversity

**Figure 1:**
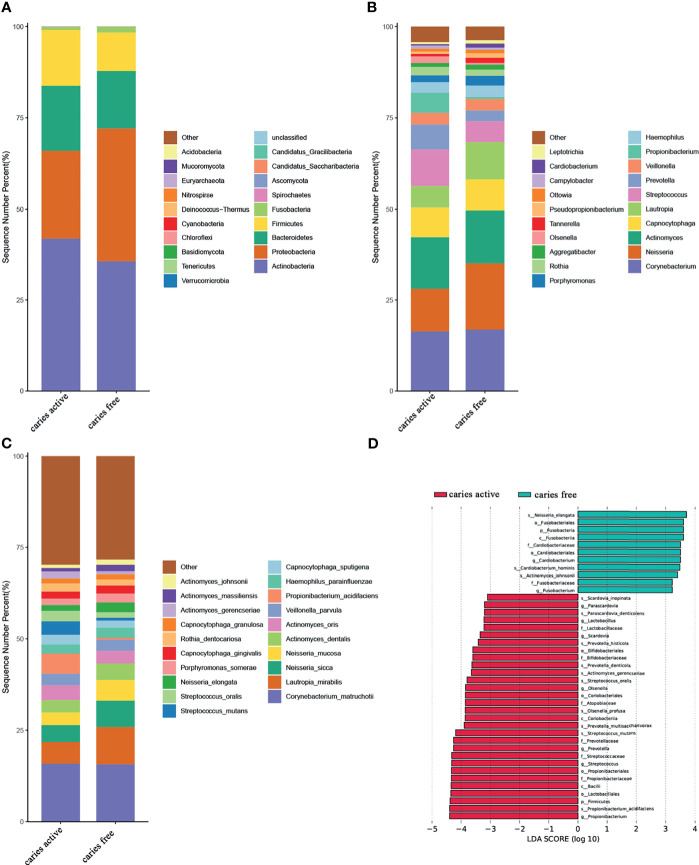
Relative abundances of plaque microbial in caries active and caries free groups. **(A–C)** Relative abundances of phyla, genera, and species from the top 20 annotated microbial taxa, respectively, are shown in a bar plot. **(D)** Significant differences in relative abundance of plaque microbial taxa between the caries active and caries-free groups, as identified by linear discriminant analysis (LDA) effect size analysis (LEFSe) (DUNN test, *p* < 0.05).

At the phylum level, *Actinobacteria*, *Proteobacteria*, *Bacteroidetes*, *Firmicutes*, and *Fusobacteria* were the most abundant taxa in both groups. Compared with the healthy controls, the caries group displayed a higher abundance of *Firmicutes* and a lower abundance of *Fusobacteria* (Dunn test, *p*<0.05) (**Figures 1A, D** and **Supplementary Table 5**).

At the genus level, *Corynebacterium*, *Neisseria*, *Actinomyces*, *Capnocytophaga*, *Lautropia*, *Streptococcus*, *Prevotella*, *Veillonella*, *Propionibacterium*, and *Hemophilus* were among the major phylotypes in these two groups (**Figure 1B**). A total of seven bacterial genera, namely, *Propionibacterium*, *Streptococcus*, *Prevotella*, *Olsenella*, *Scardovia*, *Lactobacillus*, and *Parascardovia*, were more abundant in the caries group, whereas *Fusobacterium* and *Cardiobacterium* were more abundant in the caries-free group (Dunn test, *p*<0.05) (**Figures 1B, D** and **Supplementary Table 5**).

At the species level, *Corynebacterium. matruchotii* (*C. matruchotii*), *Lautropia mirabilis* (*L. mirabilis*), *Neisseria sicca* (*N. sicca*), and *Neisseria mucosa* (*N. mucosa*) occupied a large part of the total microbial abundance in the two groups, indicating that these microbes may belong to the stable plaque microflora (**Figure 1C**). Three species were more abundant in the caries-free group, including *Neisseria elongata* (*N. elongata*), *Cardiobacterium hominis* (*C. hominis*), and *Actinomyces johnsonii* (*A. johnsonii*). Conversely, six species were found to be enriched in the caries-active group. These species included *Propionibacterium acidifaciens* (*P. acidifaciens*), *Actinomyces gerencseriae* (*A. gerencseriae)*, *Prevotella multisaccharivorax* (*P. multisaccharivorax*), *Parascardovia denticolens* (*P. denticolens*), *Streptococcus mutans* (*S. mutans*), and *Streptococcus oralis* (*S. oralis*). All these species have been reported to be associated with caries (Dunn test, *p*<0.05) (**Figures 1C, D** and **Supplementary Table 5**).

**Figure 2:**
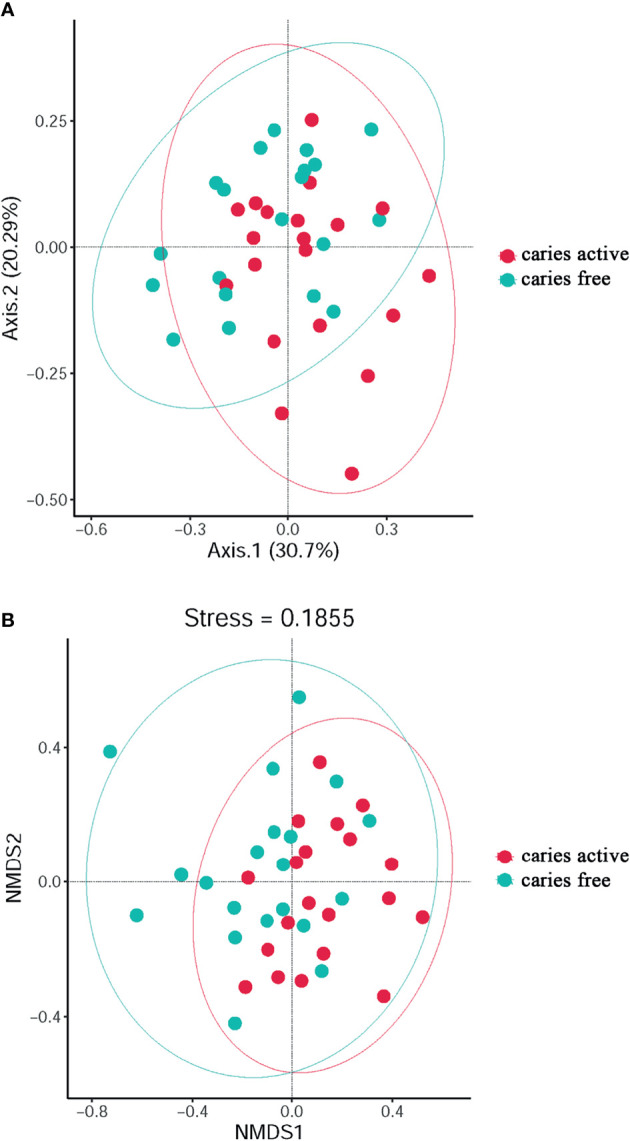
The diversity analysis between groups: PCoA **(A)** and Non-Metric Multi-Dimensional **(B)** analysis based on Bray-Curtis distances according to the abundance tables of microbial taxa at the genus level. Circles in different colors represent different groups.

PCoA (**Figure 2A**) and NMDS (**Figure 2B**) were calculated for the species used to investigate the diversity of plaque microbiome richness. The results showed that microbial diversity was similar between the caries-free and caries-active groups.

**Figure 3:**
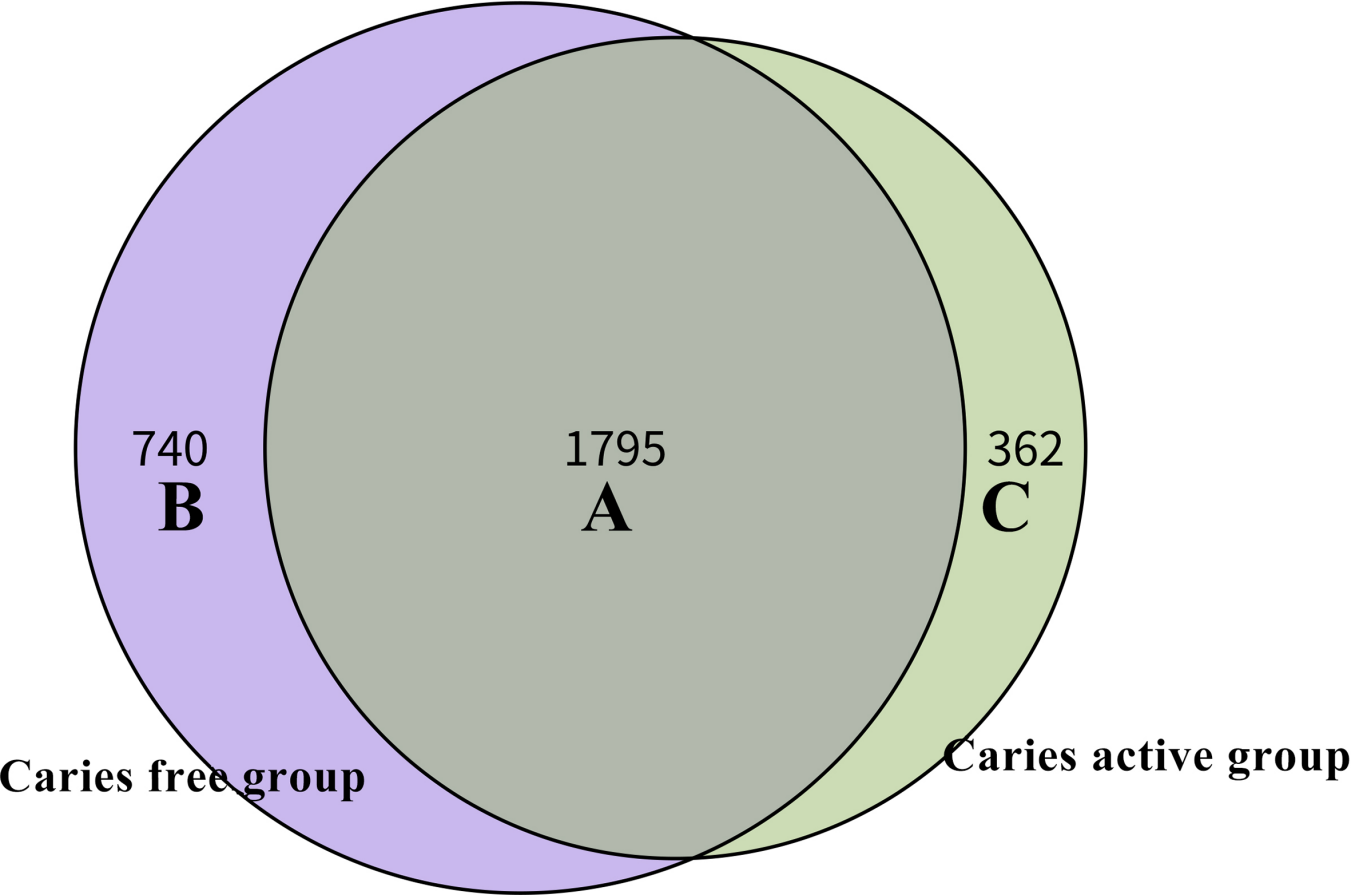
Venn diagram at the species level. The overlap region A represents the microbiome shared in all samples among caries-active and caries-free groups. Region B represented the unique taxa in caries-free controls and Region C represented the unique taxa in caries-active group.

Venn diagram was used to illustrate the core microbiome, which is defined as the taxa shared across all samples in a certain group or among groups. A total of 2,897 species were identified in the present study. Among them, 2,157 species belong to caries-active teenagers and 2,535 belong to caries-free controls. The overlap region A represents the microbiome shared in all samples among caries-active and caries-free groups. Region B (740 species) represented the unique taxa in caries-free controls and Region C (362 species) represented the unique taxa in the caries-active group (**Figure 3**).

#### Functional Characteristics of the Microbiota Between Groups

**Figure 4:**
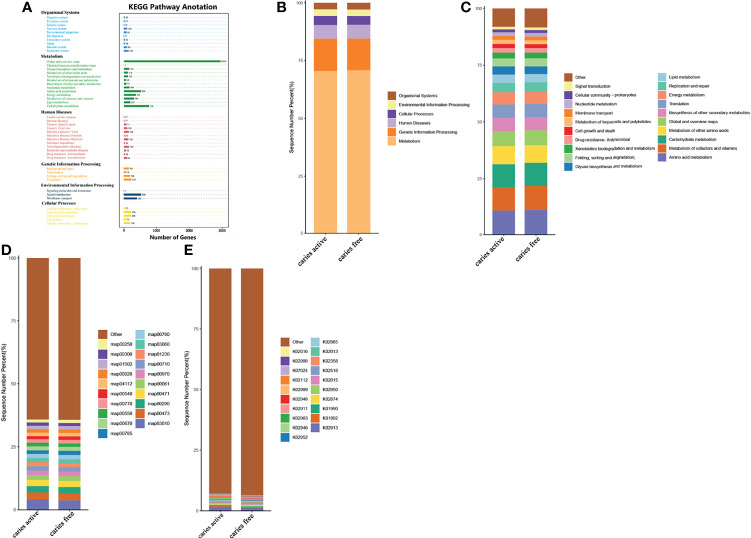
Comparing the functional characteristics of the microbiome caries active group and caries free controls. **(A)** The number of genes annotated in the Kyoto Encylopedia of Genes from the 40 samples. **(B)** The comparison of functional KEGG between the two groups at the level 1 pathway, **(C)** level 2 pathway, **(D)** level 3 pathway, and **(E)** ortholog level. The horizontal axis represents the relative abundance of annotated genes. The histograms show the top 20 annotated genes predicted in the metabolic pathways.

To investigate the functional characteristics of the microbiota between groups, we annotated the oral gene catalogs using the Kyoto Encyclopedia of Genes and Genomes database (KEGG). Six main pathways in KEGG were identified, and the most abundant pathway was the metabolism pathway, including 5,593 annotated genes (**Figure 4A**), which accounted for 70.70% of all annotated genes (**Figure 4B**). There were 57 level 2 pathways, the most abundant of which was amino acid metabolism, containing 504 annotated genes and occupying 10.70% of the total relative abundance (**Figure 4C**). Other common markers in the level 2 pathways was included in the metabolism of cofactors and vitamins and carbohydrate metabolism. The top 10 functional annotations at level 3 pathways were mostly involved in metabolic pathways (map00473: D-alanine metabolism; map00290: valine, leucine and isoleucine biosynthesis; map00471: D-glutamine and D-glutamate metabolism; map00061: fatty acid biosynthesis; map00710: carbon fixation in photosynthetic organisms; map01230: biosynthesis of amino acids; map00780: biotin metabolism), and genetic information processing (map03010: ribosome, map00970: aminoacyl-tRNA biosynthesis; and map03060: protein export) (**Figure 4D**). By performing BLAST against the KEGG Orthology (KO) database, the top 10 most abundant KOs were obtained, and they are illustrated in **Figure 4E**.

**Figure 5:**
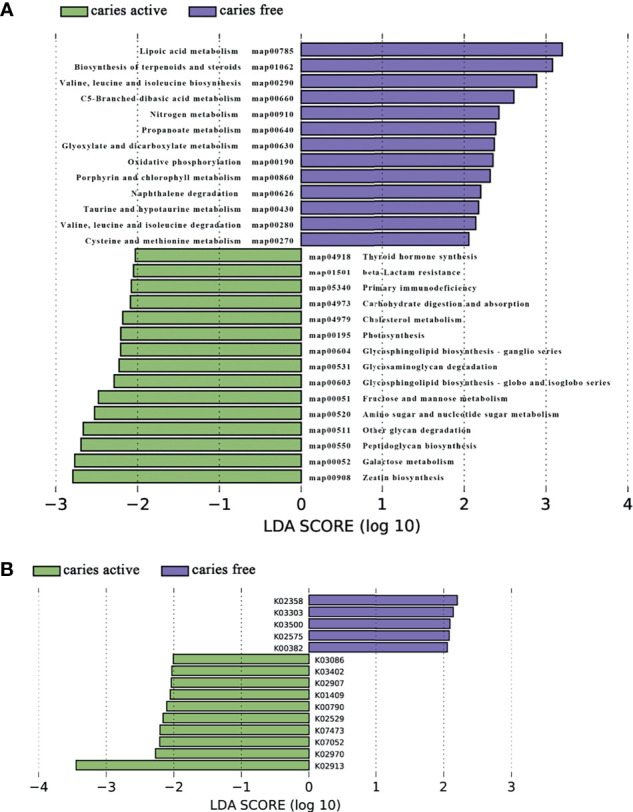
Linear discriminant analysis (LDA) effect size analysis (LEFSe) was used to identify level 3 pathways **(A)** and KOs **(B)** with significant differences in relative abundance between the caries active and caries-free groups.

**Figure 6:**
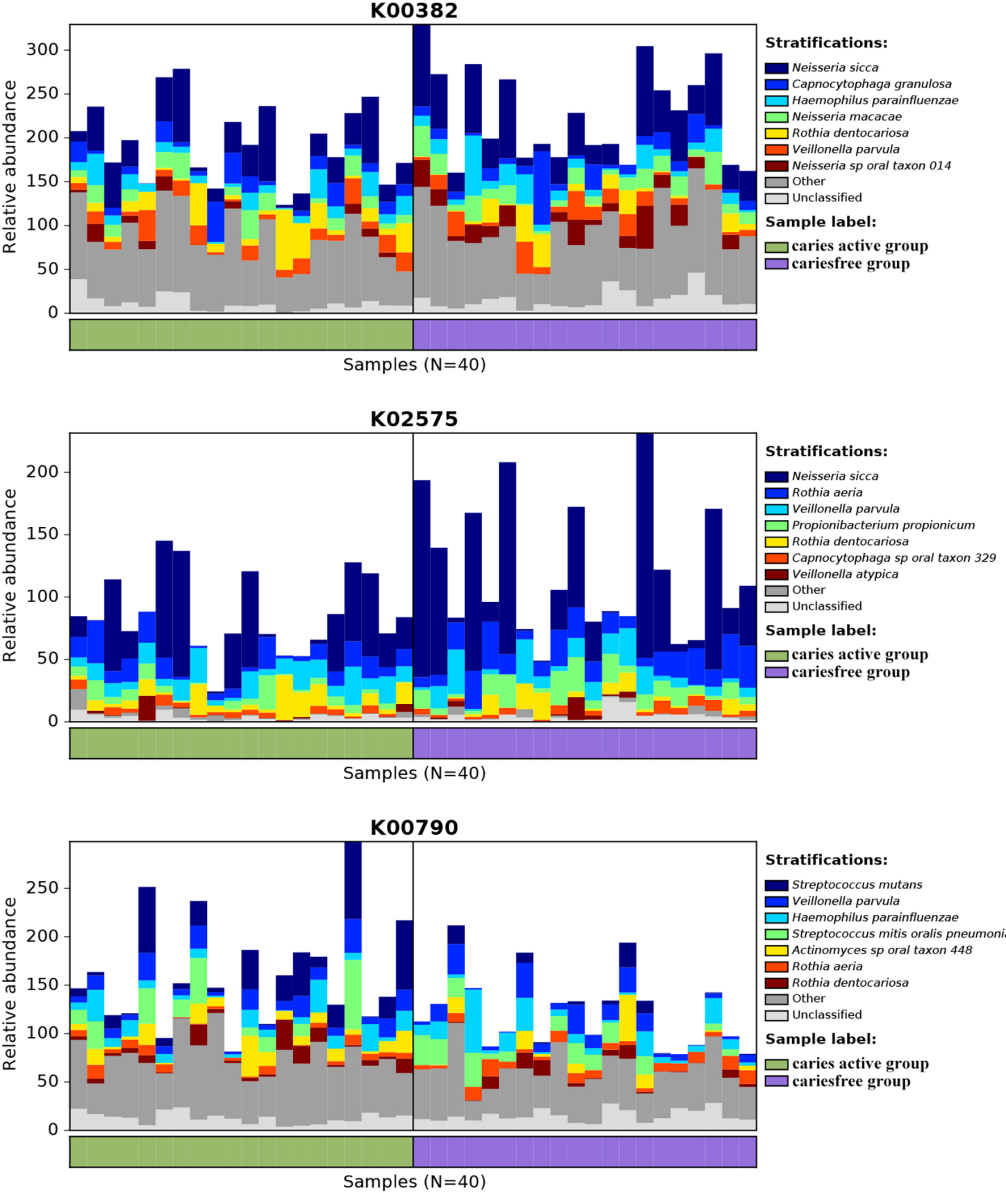
Species contributions to key functions. For each identified KO, HUManN2 was used to calculate the species contribution. In the figure are species contributions to three features correlated to dental caries.

Analysis of the gene profile revealed a significant functional difference between the two groups at each pathway level. Annotated genes that were differentially abundant to the KEGG databases revealed that significant differences were found between groups in several pathways/functions. Compared with the caries-free group, the caries-active group had a relatively increased level of nucleotide metabolism, immune diseases, lipid metabolism, metabolism of terpenoids and polyketides and decreased levels of chemical structure transformation maps, cancer-specific types, and nervous system. Differences were also identified at the third-level components of KEGG between caries-active and caries-free groups. In those components, biosynthesis of zeatin, peptidoglycan, and glycosphingolipid and metabolism of galactose, amino sugars and nucleotide sugars, fructose and mannose, and cholesterol were strongly associated with caries-active status, while metabolism of lipoic acid, C5-branched dibasic acid, nitrogen, propanoate, glyoxylate and dicarboxylate, porphyrin and chlorophyll, taurine and hypotaurine, cysteine and methionine and biosynthesis of terpenoids and steroids, valine, leucine, and isoleucine were strongly associated with caries-free status. (**Figure 5A**) We also found several KOs with significant differences in relative abundance between the caries-active and caries-free groups, and the results are shown in **Figure 5B**. K00382 participated in regulating dihydrolipoamide dehydrogenase, and the latter associated with metabolism of several components, which enriched in the caries-free group, including propanoate, glyoxylate and dicarboxylate, valine, leucine, and isoleucine (**Supplementary Data Sheet 1**), while K00790 was involved in regulating UDP-N-acetylglucosamine 1-carboxyvinyltransferase, which was related to peptidoglycan biosynthesis and amino sugar and nucleotide sugar metabolism that enriched in the caries-active group (**Supplementary Data Sheet 1**). **Figure 6** reveals the species that contribute to key functions. The results suggested that *N. sicca* contributed to metabolic changes of caries-free status, while *S. mutans* associated with metabolic changes related to caries-active status (**Figure 6**).

#### Cooccurrence Networks of Plaque Microbiome Under Caries-Active and Caries-Free Conditions

**Figure 7:**
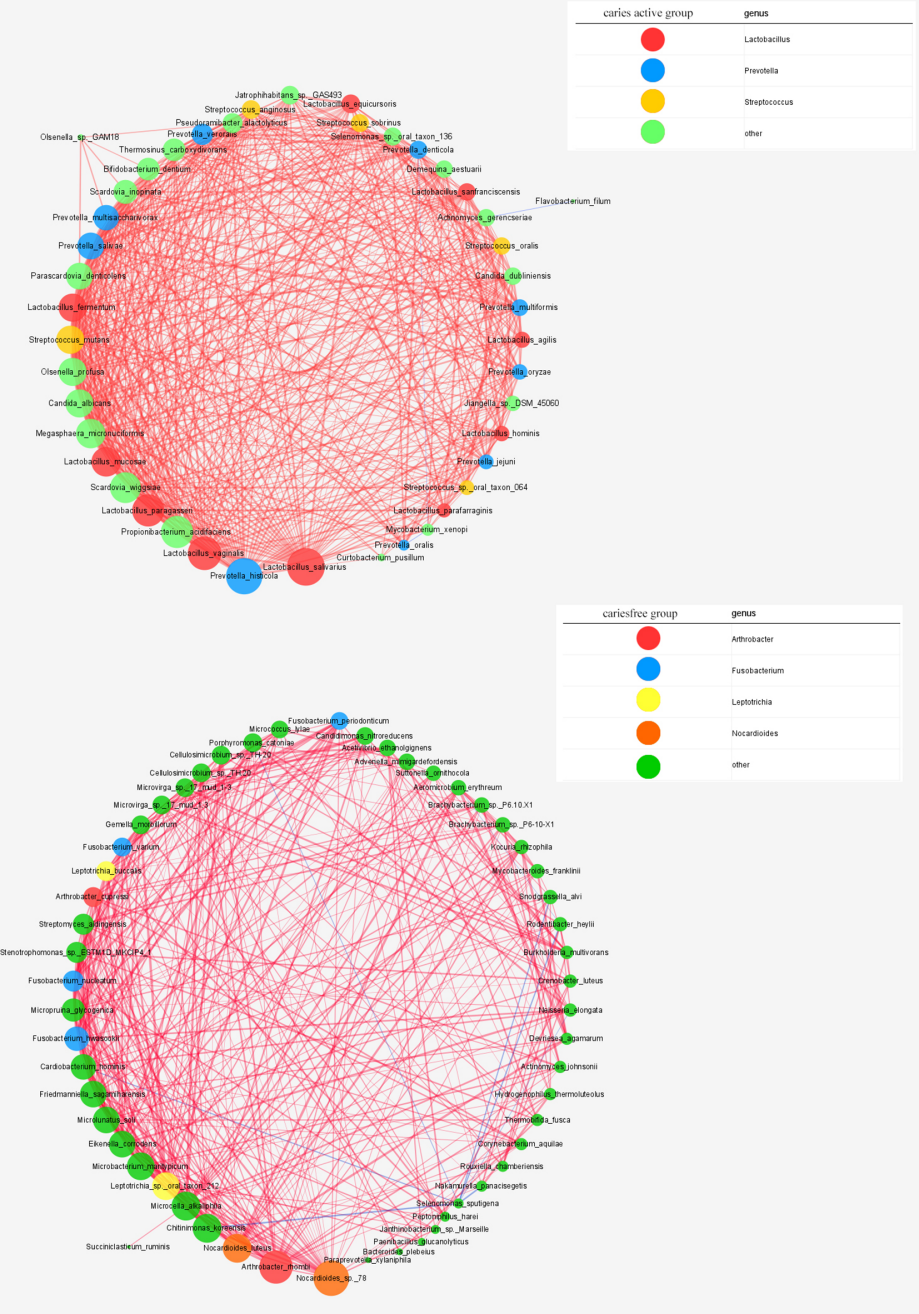
Networks in dental microbial communities under caries-active and caries free status are shown, with each microbial species and cooccurrence relationship indicated by a node and an edge, respectively. A connection (line between dots) indicates a strong (Spearman’s p >0.6) and significant (p < 0.05) correlation. The size of each node is proportional to the relative abundance. Lines between nodes indicate positive correlations (red) or negative correlations (blue). The top thress abundant genera are indicated in color.

Cooccurrence network was constructed to reveal the interbacterial interactions in dental plaque microbial communities under caries-free and caries-active conditions. The cooccurrence network of the caries-active group comprises 44 species with interbacterial interactions, as well as a network of the caries-free group containing 55 species. *Lactobacillus* spp., *Prevotella* spp., and *Streptococcus* spp. were dominant in the caries-active network, while *Arthrobacter* spp., *Fusobacterium* spp., *Leptotrichia* spp., and *Nocardioides* spp. were the predominant species in the caries-free network (**Figure 7**).

#### Host Clinical Phenotypes Associated With Some Microbial Taxa and Pathways

We performed MaAsLin to identify microbial species associated with the host clinical phenotypes. A total of 312 representative species (>0.01% of total microbial reads and present in at least 10% individuals) were included in the analysis, and a total of 13 species from 8 phyla were positively correlated with the caries states (FDR<0.05). In addition, brushing with fluoride toothpaste was negatively related to the abundance of *Bifidobacterium animalis* (FDR<0.05) (**Supplementary Table 2**). However, no significant associations were found between host clinical phenotypes and pathways profiles (**Supplementary Table 3**).

PERMANOVA revealed that caries status was significantly associated with interpersonal variation of microbial species composition (*p* = 0.008) and pathway (metacyc) profiles (*p* = 0.019), but not the main driving factor for the overall structural and functional variation of the dental plaque microbiome (*R*^2^ = 0.082 and *R*^2^ = 0.069, respectively). In contrast, the potential cofounding variables including gender, dietary habit, and brushing habits made no significant contributions to the plaque microbiome composition and pathway (metacyc) profiles (**Supplementary Table 4**).

## Discussion

Worldwide, caries is one of the most prevalent infectious diseases (Kassebaum et al., 2015), and pit and fissured surfaces are the most susceptible caries sites (Batchelor and Sheiham, 2004). Dysbiosis of the local microbiota is one of the critical variables that cause caries. In our research, dental plaque was collected from healthy pits and fissured surfaces of caries-active adolescents and caries-free adolescents to investigate microbial factors involved in susceptibility of caries. To the best of our knowledge, this study presents the first effort to investigate the effects of plaque microbes in pit-and-fissured sites on caries using metagenomics approaches. Our results revealed taxonomic and functional differences in the plaque microbiomes in the pit-and-fissure sites between the two groups. Our findings suggested that in addition to the specific anatomical structures of the pit and fissured surfaces, the significant differences in the plaque microbiome ecology also contribute to the susceptibility of pit and fissure caries.

Although the overall taxonomic compositions of the two groups were similar, several species that were enriched in the caries group were found, which can be used as candidate biomarkers for pit and fissure caries prognosis in the future. Several studies reported that higher levels of *A. gerencseriae* were related with childhood caries (Becker et al., 2002; Tang et al., 2003), indicating that *A. gerencseriae* had a positive correlation to early childhood caries. In agreement with these reports, *A. gerencseriae* was enriched in the caries group, suggesting that *A. gerencseriae* may also be an important contributor to pit and fissured surface caries of permeant teeth. Consistent with our results, previous studies have also reported that *P. acidifaciens* and *P. multisaccharivorax* can be detected in the plaques of individuals with progressive carious lesions (Wolff et al., 2013; Richards et al., 2017; Skelly et al., 2020). *P. acidifaciens* is a gram-positive anaerobic bacterium (Downes and Wade, 2009), which can produce acids and is tolerant to self-produced acids at a lower pH than that of threshold concentrations (Obata et al., 2019). These factors may contribute to the development of caries.

The importance of *S. mutans* in the occurrence and progression of caries is a recently debated subject (Banas and Drake, 2018). In our study, the mean amount of *S. mutans* in the caries-active group was 4.67 times that of the caries-free group, suggesting that *S. mutans* was significantly associated with caries. However, *S. mutans* was not identified in all subjects in the caries group but was identified in the caries-free group, which is consistent with the concept that *S. mutans* cannot sufficiently explain all cases of caries. *P. denticolens* (formerly *Bifidobacterium denticolens*) was taxonomically related to the genus *Bifidobacterium*, and the latter was considered to be an aciduric bacteria associated with caries. (Gueimonde et al., 2012). Consistent with our results, Wolff et al. (2019) also reported *P. denticolens* enriched in carious dentin samples. A previous study by de Matos BM and colleagues demonstrated that *S. mutans* and *P. denticolens* have a synergistic effect that promotes a higher pH drop than the individual pH drops, which means that the presence of both species may indicate a higher cariogenic potential (de Matos et al., 2017).

*N. elongata* belongs to the genus *Neisseria*. Previous reported data on the relationship between this genus and caries status are controversial. Several studies have suggested that the *Neisseria* genus is associated with caries (Zheng et al., 2018), while other studies believe that the *Neisseria* genus is more prominent in the caries-free group (Richards et al., 2017). The main reason for this debate was that some species in the genus *Neisseria* can metabolize sugar to lactate, and other members of this genus can metabolize lactate to lower microenvironmental acidification. In our study, *N. elongata* were identified as caries-free, consistent with Qudeimat et al. (2021). *A. johnsonii* was originally classified as the species *A. naeslundii* before its reclassification in the current species tax (Henssge et al., 2009). *A. naeslundii* is related to the balance of biofilm ecology and is always correlated with a healthy microbiota (Takahashi and Nyvad, 2008). Consistent with reported studies, we also found that *A. johnsonii* was enriched in the healthy controls. de Oliveira RVD reported that *A. johnsonii* is capable of increasing local pH by producing ammonia and alkali and can lead to a decrease in lactic acid production when interacting with *S. mutans* (de Oliveira et al., 2020). Our results suggest that *C. hominis* was enriched in caries-free adolescents and that its connections with caries have not been reported. *C. hominis* is a member of the genus *Cardiobacterium* and is notorious for causing apparent culture-negative endocarditis. Information regarding the association between *C. hominis* and oral disease is scarce. Çelik et al. (2021) reported that *Cardiobacterium* were more abundant in plaque samples of caries-free adults with black stain. Another report showed that *C. hominis* was more prevalent in periodontal health than in periodontitis patients (Colombo et al., 2009).

Similar to the results of Wang et al. (2019), the cooccurrence network in our study suggested that *Lactobacillus* spp., *Streptococcus* spp., and *Prevotella* spp. were the main genera under the caries-active status. However, in the caries-free status, our results showed that *Arthrobacter* spp. and *Fusobacterium* spp. were the dominant genera, different from results reported by Wang et al. (2019). Two reasons contributed to this inconsistency, the first one is different research population, another one is different sample source.

To comprehensively understand the plaque microbial community, we analyzed not only the composition and structure of the community but also the function of the community. Our results suggested that the overall functional gene composition was similar between the two groups. When comparing the relative abundance of functional classifications, we discovered that the different metabolic functions were enriched in separate groups. Those enrichment represented that the microbial metabolism of the plaque bacterial community was vigorous. However, the differences of relative abundance between the two groups in various functional levels and prompting the diverse functions of the biofilms were significant. Carbohydrates have been considered one of the main causes of dental caries because cariogenic bacteria can metabolize carbohydrates to generate organic acids and extracellular polysaccharides (EPS). Organic acids could produce a low pH microenvironment in biofilms with the help of an EPS-rich matrix. The low pH microenvironment could cause enamel demineralization and impose stress on the biofilm, leading to microbial community transfer to a cariogenic state, which is mainly composed of acidic and aciduric bacteria (Paes et al., 2006). Therefore, the lower pH microenvironment caused by carbohydrate metabolism plays a significant role in the occurrence and development of caries. We found that the genes related to carbohydrate digestion and absorption presented higher relative abundance levels in the caries group than the caries-free group, indicating that the microbiome on the pit and fissure surfaces of the caries-active patients had a greater capacity of carbohydrate digestion and absorption, which could provide more carbohydrates for the metabolism of biofilms. The genes associated with nitrogen metabolism were enriched in the caries-free group, indicating that the microbiome of the healthy controls had a higher ability to metabolize nitrogenous compounds. Bacterial catabolism of nitrogenous compounds is important, as the end-products can raise plaque pH, which could in part explain why these people are not prone to caries (Rogers, 1990).

## Conclusion

In conclusion, the current study suggested that the plaque microbiomes of the pit and fissure site exhibited significant differences in relative abundance of several species and genes between caries-active adolescents and caries-free controls (Dunn test, *p <* 0.05). Six species enriched in the caries-active group were identified, and they can be used as caries candidate biomarkers in the future. We found that the most abundant genes identified in the two groups were associated with carbohydrate metabolism, metabolism of cofactors, and vitamins and amino acid metabolism. The potential functional differences between the groups were mainly distributed in metabolic pathways involving nitrogen metabolism and carbohydrate digestion and absorption. There was an obvious limitation in the present study that the sample size was small and allows only preliminary findings. Given this, one should be cautious and not overextend the implications of the findings. In the future, larger-scale studies are needed to confirm and validate our findings. Host immunological and environmental factors and the potential connections between oral bacteria and these factors during caries should also be taken into consideration.

## Data Availability Statement

The data presented in the study are deposited in the NCBI repository, accession number PRJNA766357 and can be accessible with the following link: https://www.ncbi.nlm.nih.gov/sra/PRJNA766357.

## Ethics Statement

The studies involving human participants were reviewed and approved by the Ethical Committee of the Hospital of Stomatology, Sun Yat-sen University, in Guangzhou, China. Written informed consent to participate in this study was provided by the participants’ legal guardian/next of kin.

## Author Contributions

LP and YW contributed to the conception and design and drafted and critically revised the manuscript. YY contributed to data acquisition and analysis and critically revised the manuscript. YZ contributed to the design and critically revised the manuscript. QZ and HL contributed to the conception and design and drafted and critically revised the manuscript. All authors contributed to the article and approved the submitted version.

## Funding

This work was supported by the Young Scientist Fund of the National Natural Science Foundation of China (grant no. 81903345).

## Conflict of Interest

The authors declare that the research was conducted in the absence of any commercial or financial relationships that could be construed as a potential conflict of interest.

## Publisher’s Note

All claims expressed in this article are solely those of the authors and do not necessarily represent those of their affiliated organizations, or those of the publisher, the editors and the reviewers. Any product that may be evaluated in this article, or claim that may be made by its manufacturer, is not guaranteed or endorsed by the publisher.
